# Patient Attitudes and Their Awareness Towards Skin Cancer–Related Apps: Cross-Sectional Survey

**DOI:** 10.2196/13844

**Published:** 2019-07-02

**Authors:** Theresa Steeb, Anja Wessely, Sebastian Mastnik, Titus Josef Brinker, Lars Einar French, Anne-Charlotte Niesert, Carola Berking, Markus Vincent Heppt

**Affiliations:** 1 Department of Dermatology and Allergy University Hospital LMU Munich Munich Germany; 2 Department of Dermatology University Hospital Heidelberg University of Heidelberg Heidelberg Germany

**Keywords:** skin cancer, melanoma, mobile applications, telemedicine, awareness, patient education

## Abstract

**Background:**

In the emerging era of digitalization and electronic health, skin cancer–related apps represent useful tools to support dermatologic consultation and examination. Yet, little is known about how patients perceive the value of such apps.

**Objective:**

The aim of this study was to investigate patient attitudes and their awareness toward skin cancer–related apps.

**Methods:**

A cross-sectional study including 200 patients from the oncological outpatient unit was conducted at the University Hospital (LMU Munich, Germany) between September and December 2018. Patients were asked to complete a self-administered questionnaire on the popularity and usefulness of health-related and skin cancer–related apps. A descriptive analysis was performed with the expression of categorical variables as frequencies and percentages. For continuous variables, the median and range were indicated. Contingency tables and chi-square tests were performed to investigate associations between sociodemographic data and selected items of the questionnaire.

**Results:**

A total of 98.9% (195/197) of patients had never used skin cancer–related apps or could not remember. In 49.7% (93/187) of cases, patients were unsure about the usefulness of skin cancer apps, whereas 42.6% (78/183) thought that skin cancer apps could supplement or support the professional skin examination performed by a physician. However, 47.9% (90/188) were interested in acquiring more information by their dermatologists about skin cancer apps. Young age (*P*=.002), male gender (*P*=.02), a previous history of melanoma (*P*=.004), and higher educational level (*P*=.002) were significantly associated with a positive attitude. Nevertheless, 55.9% (105/188) preferred a printed patient brochure on skin cancer to downloading and using an app.

**Conclusions:**

The experience and knowledge of skin cancer–related apps was surprisingly low in this population, although there was a high general interest in more information about such apps. Printed patient brochures were the preferred information source.

## Introduction

Nonmelanoma skin cancer (NMSC) is the most common malignancy in fair-skinned population groups. In Germany, the incidence was 221,800 new cases in 2014 [1]. About 77% of all NMSCs are basal cell carcinoma and 22% are squamous cell carcinoma [2]. Melanoma arises from the melanocytes of the skin and accounted for 21,220 new cases in Germany in 2014 [1]. It is the fifth most common malignancy among cancer patients. The incidence of melanoma has been steadily increasing worldwide [3,4]. As a result of the increasing incidence of skin cancer [2,5] as well as the approval of new treatment regimens for advanced skin cancer, such as immune checkpoint blocking antibodies with unprecedented efficacy rates [6,7], the need for detailed patient information and education, for example, on potential adverse events under immunotherapy, is rising enormously. A survey among melanoma patients in German skin cancer centers including 67% patients with metastatic melanoma showed that more than half of the patients wished to receive advice on information resources that they can use outside the clinic to inform themselves [8]. Additionally, recent research suggests that the information-seeking behavior of melanoma patients and the resources they use have been changing with the accessibility of modern media [9-11]. Hence, in response to the increasing incidence and the growing demand on condition-related education, skin cancer–related and preventive smartphone apps have been launched successfully both for patients [12-14] and for health care professionals [15]. Most of the apps are easily accessible and address various topics, such as the detection of skin cancer via computer-based algorithms, self-examination or telemedicine, the tracking of skin changes, or the prevention of sunburns or skin cancer.

Physicians still serve as the primary information source for patients diagnosed with any cancer entity [16,17]. However, as physicians have limited time for comprehensive education [8,18], many patients tend to use further information sources to compensate for their informational deficits [19,20]. Skin cancer–related smartphone apps represent a useful supportive information tool to complement the physician’s consultation. They have high potential to improve participatory decision making and informed consent. In this paper, we report the results of a cross-sectional study to investigate the dissemination of skin cancer–related smartphone apps among patients, patient attitudes toward the use of skin cancer–related smartphone apps, and the association between sociodemographic variables and the usage of such apps.

## Methods

### Study Design and Ethics Approval

A cross-sectional study that included patients from the oncological outpatient clinic of the Department of Dermatology and Allergy of the University Hospital of Munich was conducted between September and December 2018. This study was approved by the institutional review board of the University Hospital (LMU Munich) on May 4, 2018 (approval number 18-336 UE). We closely adhered to the Strengthening the Reporting of Observational Studies in Epidemiology statement for cross-sectional studies for the reporting of this study [21,22].

### Setting and Participants

The oncological outpatient unit mainly focuses on the treatment and surveillance of patients with a previous diagnosis of any type of skin cancer undergoing follow-up care. Thus, the majority of the study population had been diagnosed with skin cancer before the assessment. All adult patients (aged 18 years or older) presenting at the unit were asked to complete a 2-page questionnaire either by a physician (SM) or a study nurse. Participation was voluntary, and all participants gave verbal informed consent before completing the questionnaire. Refusals were not documented, and no incentives were provided. Relatives or accompanying persons were excluded from the study. Each patient was allowed to participate only once in the survey (cross-sectional design).

### Survey

**Figure 1:**
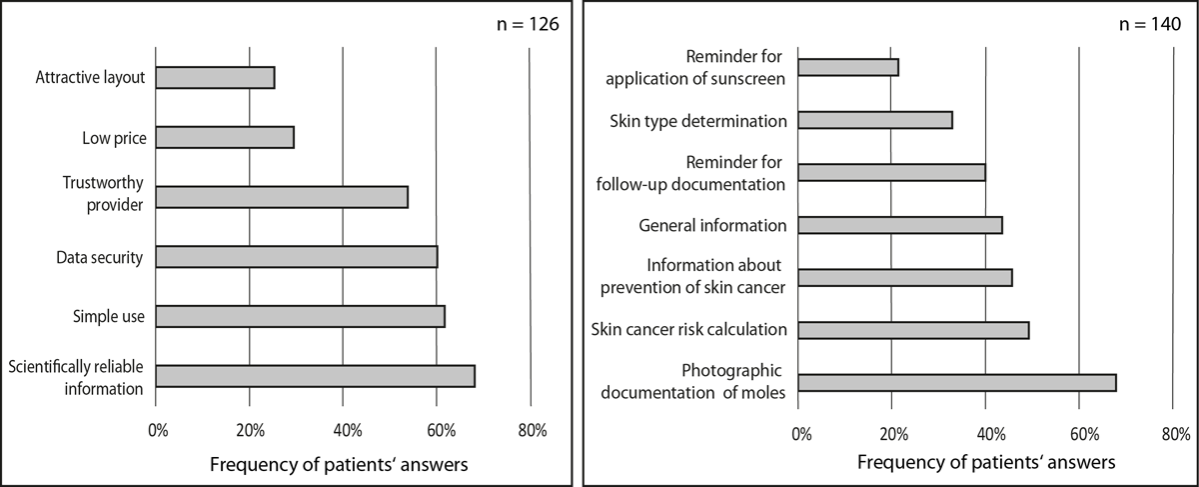
The frequencies of patients’ specific answers regarding the preferred features in health-related apps in general (left) and skin cancer–related apps (right).

As no validated survey tools existed for the objective of our study, the questionnaire was developed de-novo based on a literature review and dermato-oncological expert consulting, including questions on skin cancer–related smartphone apps and basic demographic information (age, gender, and highest level of education). In a multiple-choice question format, patients were asked about the reason of presenting to the unit at the day of the assessment and whether they had already been diagnosed with skin cancer before. Other questions addressed the patients’ previous use of health-related apps and, specifically, skin cancer–related apps and their relevance for skin cancer detection as well as concerns regarding digital security. These questions were dichotomous; however, patients could also state that they were unsure. The questions are presented in Figure 1. The full questionnaire can be obtained from the Multimedia Appendix 1. The questionnaire was pretested by independent researchers (TJB and A-CN) and patients without skin cancer for clarity and comprehension. On the basis of their suggestions, the questionnaire was revised to the final form. Completed questionnaires were sequentially numbered for data entry purposes but were not linked to any identifying patient information to assure irreversible anonymity.

### Data Analysis

We calculated an estimated sample size of n=197 required for this descriptive study with an alpha error of 5%, a power of 80%, a CI of 95% and a relevant effect strength of 20%. The calculation was based on the item “Do you find the use of skin cancer apps useful for patients?” as we hypothesized that this question would be the most appropriate and a global indicator for the patient attitude toward skin cancer apps. The effect strength of 20% was a conservative estimate based on a previous study which was performed in the Munich area where approximately 25% did not own a mobile device or had access to a personal computer only [23]. For statistical analysis, the categorical variables were expressed as frequencies and percentages and were compared using the chi-square tests. For continuous variables, the median and range were used. A 2-sided *P* value <.05 was considered statistically significant. Statistical analyses were conducted with SPSS (IBM SPSS Statistics version 25, IBM Corporation).

## Results

### Baseline Characteristics of the Study Population

A total of 200 patients were included, 34.4% (67/195) of whom had an appointment for skin cancer treatment; 49.7% (97/195) underwent skin cancer screening, and 20.0% (39/195) had a follow-up appointment; 6.2% (12/195) of the patients presented because of a suspicious mole (multiple answers possible, hence values do not sum up). The majority of patients had already been diagnosed with skin cancer (173/193), most of them with melanoma (131/186, 70.4%), followed by basal cell carcinoma (32/186, 17.2%). The median age was 66 (range 20-91) years, and 62.7% (121/193) were males (Table 1).

**Table 1:** Baseline characteristics of the study population.

Characteristics			Values
**Sex (n=193), n (%)**			
	Female		72 (37.3)
	Male		121 (62.7)
**Age (years; n=190)**			
	Median (range)		66 (20-91)
	Mean (standard deviation)		62.63 (15.56)
**Education (n=183), n (%)**			
	**High level of education**		
		University degree	41 (22.4)
		General higher education entrance qualification	32 (17.5)
	**Middle to low level of education**		
		Secondary school leaving certificate	52 (28.4)
		Lower secondary school leaving certificate	52 (28.4)
	**Other**		
		Other degree	4 (2.2)
		No degree	2 (1.1)
**Reason for appointment (n=195, multiple answers possible), n (%)**			
	Skin cancer screening		97 (49.7)
	Skin cancer treatment		67 (34.4)
	Follow-up visit		39 (20.0)
	Suspicious mole		12 (6.2)
**Previous diagnosis of skin cancer (n=193), n (%)**			
	No		20 (10.4)
	Yes		173 (89.6)
**Type of skin cancer (n=186, multiple answers possible), n (%)**			
	Melanoma		131 (70.4)
	Basal cell carcinoma		32 (17.2)
	Squamous cell carcinoma		17 (9.1)
	Other (including actinic keratosis and Merkel cell carcinoma)		19 (10.2)

### Previous Experience With Health Apps

A total of 66.7% (130/195) of patients were owners of a smartphone and 31.8% (62/195) of a tablet device. Additionally, 8.7% (17/195) reported to also use other devices such as wearables. When asked about previous experiences with health-related apps, 8.5% (17/199) stated that they had previously made use of such apps, whereas the overwhelming majority (180/199, 90.5%) denied or was unsure about it (2/199, 1.0%). Apps that had already been used by the patients were predominantly health-tracking apps offered by Apple and Android or the fitness-tracking app, Runtastic. Others included diet apps such as Weightwatchers and apps provided by health insurance companies (GesundheitsApp der Gothaer Krankenversicherung AG) and a skin cancer–related app (SkinVision).

Most patients (86/126, 68.3%) rated scientifically reliable information as the most important feature for health-related apps, followed by user convenience (76/126, 60.3%) and data security (76/126, 60.3%). For 54.0% (68/126) of patients, credibility of the app provider was important; 29.6% (37/125) and 25.4% (32/126) considered a low price and an attractive layout as critical, respectively (Figure 1).

### Attitude Toward the Use of Skin Cancer–Related Apps

Only 1% (2/197) of the patients had already used skin cancer–related apps, namely the app, SkinVision. The majority of patients had never used skin cancer apps (189/197, 95.9%) or did not remember a previous usage (6/197, 3.0%).

**Figure 2:**
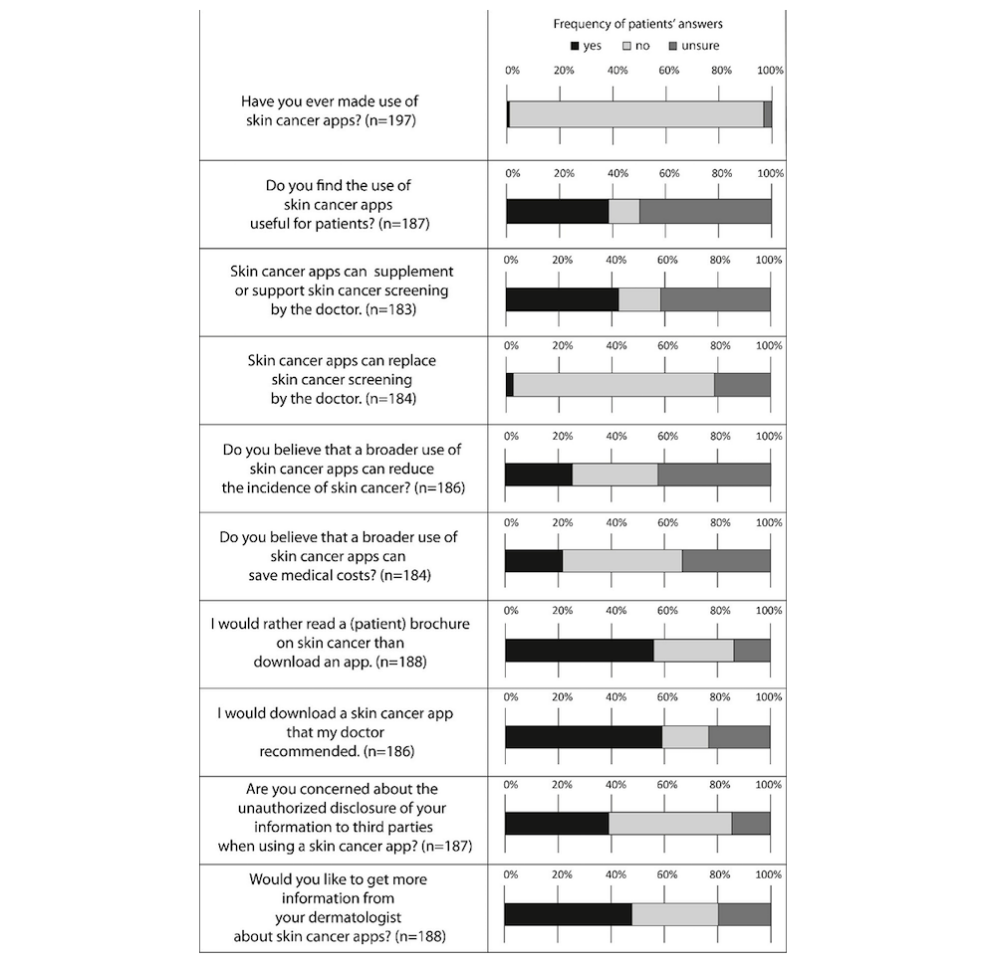
The frequencies of patients’ specific answers regarding their attitude toward skin cancer apps.

Half of the patients (93/187, 49.7%) were unsure about the usefulness of skin cancer apps for patients, whereas 38.5% (72/187) thought that such apps are useful for patients; 42.6% (78/183) voted that skin cancer apps can supplement or support professional skin cancer screening by a physician, whereas 41.5% (76/183) were unsure. The majority figured that skin cancer apps cannot replace skin cancer screening performed by a physician (cannot be replaced: 76.1% [140/184] and unsure: 21.2% [39/184]). Less than half of the patients (83/184, 45.1%) thought that a broader usage of skin cancer apps can reduce medical costs (33.2% [61/184] were unsure) and did not agree with the statement that skin cancer apps can contribute to the reduction of the incidence of skin cancer (disagree: 32.3% [60/186] and unsure: 42.5% [79/186]). Nevertheless, nearly half of the patients (90/188, 47.9%) were interested in acquiring more information from their dermatologist about skin cancer apps (Figure 2).

Interestingly, 55.9% of patients (105/188) preferred a printed patient brochure on skin cancer to downloading and using an app; 39.0% (73/187) of patients had concerns about the unauthorized disclosure of information to third parties, 46.5% (87/187) did not have concerns, and 14.4% (27/187) were unsure. However, 59.1% of the patients (110/186) would download a skin cancer app recommended by their physician.

We also asked the patients which features they find important for skin cancer apps. The majority reported the documentation of moles with photos to be important (95/140, 67.9%), followed by individual skin cancer risk calculation (69/140, 49.3%), information on the prevention of skin cancer (64/140, 45.7%), general information on skin cancer (61/140, 43.6%), and reminders to take regular photos for a follow-up examination (56/140, 40.0%). One-third (46/140, 32.9%) found the individual determination of the skin type to be important, whereas 21.4% (30/140) favored reminders for the re-application of sunscreen (Figure 1).

### Association Between Sociodemographic Data and Attitude Toward Skin Cancer Apps

There was a statistically significant association between owners of a tablet and the previous use of a health-related app (*P*=.01), that is, patients who were owners of a tablet device were more likely to already have used health-related apps.

With regard to the preferred features of skin cancer apps, patients with a history of melanoma found the photographic documentation (*P*=.04), reminder function for follow-up documentation (*P*=.03), and the possibility for a risk calculation (*P*=.002) more important than those without a previous diagnosis of melanoma. Furthermore, melanoma patients were more interested in acquiring more information on skin cancer apps (*P*=.02), and they would also download an app that is recommended by their physician (*P*=.004). Men were generally more willing to download an app that has been recommended by their physician than women (*P*=.02).

Besides, patients aged >61 years rather did not think that skin cancer apps can replace the physician in comparison to those under the age of 61 years (*P*=.02). In contrast to this, older people were more likely to think that the usage of skin cancer apps can contribute to saving medical costs (*P*=.008). People aged over 61 years in our sample would rather read a printed brochure on skin cancer than download an app (*P*<.001). In contrast, patients under the age of 61 years would download an app that has been recommended by their physician (*P*=.002), and they were more interested in acquiring additional information on skin cancer apps (*P*=.01). People with low-middle level of education rather agreed that skin cancer apps can replace the physician (*P*=.008). They would also rather read a brochure than download an app (*P*=.003). Higher educated patients would rather agree in downloading an app that has been recommended by their physician (*P*=.002).

Patients agreeing with the statement that skin cancer apps are useful for patients also thought that skin cancer apps can supplement or support skin cancer screening by the physician (*P*<.001). However, they disagreed with the statement that apps can replace skin cancer screening by a physician (*P*=.001). Additionally, they were more likely to think that skin cancer apps can contribute to the reduction of the incidence of skin cancer (*P*<.001). Interestingly, they did not think that such apps can contribute to saving medical costs or were unsure about it (*P*<.001). Patients rating skin cancer–related apps as useful preferred downloading an app to reading a brochure (*P*=.002). They were also more likely to download an app that has been recommended by their physician (*P*<.001) in comparison to patients who do not think that the usage of apps is reasonable. Yet, those who thought that skin cancer apps are useful were interested in gaining more information related to this topic (*P*<.001).

## Discussion

### Principal Findings

This cross-sectional study was designed to characterize patient attitudes toward skin cancer–related apps. Surprisingly, we observed a substantial lack of patients’ knowledge about the availability and usability of health-related apps in general. Our results contrast sharply with the recent perception of health-related apps among health care professionals in the emerging era of electronic health and digitalization [24,25]. Only a minority of study participants were aware of the existence of skin cancer apps to support their skin examination. As we surveyed a population in which most patients had been previously diagnosed with skin cancer, we assume that this lack of awareness is even higher in the general population and persons who have never faced a diagnosis of skin cancer. However, our survey also identified subgroups that seemed more amenable to the usage of apps. In particular, patients younger than 61 years and men were significantly more frequently interested in acquiring further information and indicated that they would download an app recommended by their physician. A similar trend was registered with regard to the level of education, as higher educated patients were more willing to use and download an app than those with lower to middle education. Interestingly, this association has also been detected in other studies [26,27]. However, analyzing a possible correlation between educational level and app usage was not reasonable for this study, as only 2 out of 197 study participants had ever used skin cancer–related apps.

Nevertheless, our results underline that the majority of patients and particularly those who were older than 61 years remain skeptical about the usage of skin cancer apps, which is in accordance with a survey conducted in cancer patients regarding general app-assisted cancer care [23]. Various reasons for this negative attitude are conceivable. Patients in our sample were more likely to think that skin cancer apps can contribute to saving medical costs on the one hand but cannot replace professional skin examination on the other hand. This ambivalence might reflect a general fear that the apps have mainly been developed to reduce costs by replacing physicians. This hypothesis would fit well with the results of a previous survey in which the wish for personal contact with the treating physician was among the most common obstacles for not using medical apps [23].

A further reason for the skepticism may be a lack of capable devices and general concerns regarding technical issues, in particular among the elderly. In comparison to the overall German population where 79% were estimated to own at least one smartphone in 2016 [28], only 66.7% (130/195) of our study population were smartphone owners. We deem this value representative for the regional population of the Munich area as it is in line with 69.6% of mobile device users in a cross-sectional study that was conducted at several oncological departments of the hospital of the Technical University of Munich [23]. The deviation from the German population might be explained by the fact that the patients in our sample were much older than the overall population in Germany (median age: 66 years vs 45.9 years) [29]. In this context, it still remains remarkable that only 2 patients had ever used a skin cancer–related app before, although more than two-thirds of all participants owned a smartphone and nearly one-third, a tablet. However, despite their lack of personal experience, almost 40% thought that these apps are generally helpful, which contrasts with the low level of awareness.

These results imply that there is an urgent need for more education and information of both skin cancer apps and the usage of electronic devices in general. Print media offer advantages as they are readable without any additional equipment. Indeed, most patients in our study preferred to read a printed patient brochure for education to downloading and using a skin cancer app. This is consistent with a previous report by Brütting et al who surveyed melanoma patients from 27 German skin cancer centers and found that the majority used the internet and booklets as their preferred source of information [30,31]. The probability of rating the internet as an important information source was 2.2 times higher in melanoma patients aged ≤55 years [30]. However, an evaluation of German booklets has shown that most of them are of medium quality and difficult to read owing to incomplete reporting and insufficient meta-information [32].

Melanoma patients preferred distinct features in skin cancer apps compared with nonmelanoma patients. The most important ones included photographic documentation, reminder for follow-up documentation, and the possibility for a risk calculation. We assume that this subgroup of patients is generally more aware of the importance of regular self-examination and a consequent follow-up of suspicious lesions because of their personal experience and medical history [33]. Photographic documentation of suspicious lesions and intelligent algorithms for data analysis can assist in the self-detection and follow-up of skin cancer. In combination with deep neural networks, algorithms were even reported to outperform dermatologists in the detection of benign lesions [34,35]. Additionally, melanoma patients were more interested in acquiring further information about skin cancer apps and eager to download an app that has been recommended by their physician. We hypothesized that the melanoma subgroup was younger and therefore more familiar with the use of new technologies. However, there was no significant association between these 2 variables.

### Limitations

We are aware that this study has several limitations. The sample comprised 200 patients presenting to the oncological outpatient unit. First, the sample size of this study is relatively small and, second, it was not sampled in a random fashion but depending on the availability of patients. Thus, the results presented here may not be fully generalizable to the general population and are at risk for sampling bias.

### Conclusions

Altogether, this cross-sectional study demonstrates that the awareness and popularity of skin cancer–related apps is still low. A high level of education, young age, male gender, and a history of melanoma were important factors for a positive attitude. To fully exploit the potential of those apps, physicians may encourage their use in these subgroups and propagate their use as a supplementary information source. However, patient brochures remain the preferred information source for skin cancer patients until now.

## Appendix

Multimedia Appendix 1 Questionnaire about patients’ attitude toward skin cancer–related apps (in German language).

## Abbreviations

NMSC: nonmelanoma skin cancer
