# Supplementary material for: Patient Attitudes and Their Awareness Towards Skin Cancer–Related Apps: Cross-Sectional Survey
Source: JMIR Mhealth Uhealth. 2019 Jul 2;7(7):e13844. doi: 10.2196/13844 (PMC6632106; doi:10.2196/13844)
Supplement: Multimedia Appendix 1 [file mhealth_v7i7e13844_app1.pdf]

Befragung zum Stellenwert von (Smartphone-) Apps in der Dermatologie und Hautkrebsvorsorge

Sehr geehrte Damen und Herren,

zunächst möchten wir Ihnen für Ihre Bereitschaft danken, an dieser Befragung teilzunehmen. Im folgenden Fragebogen werden Angaben zu Ihrer Meinung bzw. Erfahrung mit hautkrebsspezifischen Apps erhoben. Die Beantwortung des Fragebogens ist freiwillig und wird nur wenige Minuten in Anspruch nehmen. Lesen Sie sich die Fragen bitte aufmerksam durch und kreuzen Sie die für Sie zutreffenden Antworten an.
Sollten Sie Fragen haben, können Sie sich über die onkologische Ambulanz an Herrn Mastnik wenden. Ihre Angaben werden selbstverständlich vertraulich behandelt und lediglich in anonymisierter Form verarbeitet.

Bitte geben Sie die ausgefüllten Fragebögen im Briefkasten vor der onkologischen Ambulanz oder in der Anmeldung der onkologischen Ambulanz ab. Vielen Dank!

Mit freundlichen Grüßen

Carola Berking

Prof. Dr. med. Carola Berking

Oberärztin
Onkologische Ambulanz
Klinik und Poliklinik für Dermatologie und Allergologie
Klinikum der Universität München
Campus Innenstadt
Frauenlobstr. 9-11
80337 München

Studienärzte:

Dr. med Markus Heppt
✉ Markus.Heppt@med.uni-muenchen.de

Sebastian Mastnik
✉ Sebastian.Mastnik@med.uni-muenchen.de

Was ist der Grund für Ihren heutigen Besuch in der Hautklinik?

Hautkrebsscreening:	☐ Ja	☐ Nein
Hautkrebsbehandlung:	☐ Ja	☐ Nein
Auffälliges Muttermal:	☐ Ja	☐ Nein

Anderer Grund: ____

Hatten Sie bereits einmal Hautkrebs in Ihrer Krankengeschichte?

☐ Ja ☐ Nein ☐ Weiß nicht

<u>Wenn ja:</u> Welche Art?

☐ Melanom („schwarzer Hautkrebs“)
☐ Basalzellkarzinom (Basaliom)
☐ Plattenepithelkarzinom(Spinaliom)
☐ Andere:__________________

Besitzen Sie derzeit eins der folgenden Geräte (Mehrfachantworten möglich)?

☐ Smartphone (internetfähiges Handy)
☐ Tablet (z.B. „iPad“)
☐ Andere:______________________
☐ Nein
☐ Weiß nicht

Haben Sie schon einmal von gesundheitsbezogenen (health) Apps Gebrauch gemacht?

☐ Ja ☐ Nein ☐ Weiß nicht

<u>Falls ja:</u> Von welchen?

____

Was finden Sie bei gesundheitsbezogenen Apps am wichtigsten? (Mehrfachantworten möglich)

☐ ansprechende Gestaltung („Layout“)
☐ wissenschaftlich fundierte Informationen
☐ vertrauenswürdige Anbieter
☐ kostenlos / niedriger Preis
☐ einfache Bedienbarkeit
☐ Sicherheit der Informationen/Datensicherheit
☐ Sonstiges: ___________________________

Haben Sie schon einmal von Hautkrebs-Apps Gebrauch gemacht?

☐ Ja ☐ Nein ☐ Weiß nicht

<u>Falls ja:</u> Bitte um Angabe der App:_____________________

Finden Sie den Einsatz von Hautkrebs-Apps für Patienten sinnvoll?

☐ Ja ☐ Nein ☐ Weiß nicht

Welche Funktionen finden Sie bei Hautkrebs-Apps wichtig (Mehrfachantworten möglich)?

☐ Fotografische Dokumentation der Muttermale
☐ Erinnerung zur Folgedokumentation
☐ Hautkrebs-Risikoberechnung durch Einschätzung eines fotografierten Muttermals durch einen computerbasierten Algorithmus
☐ Hauttypbestimmung
☐ Informationen zur Prävention von Hautkrebs
☐ Allgemeine Informationen zu Hautkrebs
☐ Erinnerung zum Auftragen von Sonnencreme

Hautkrebs-Apps können das Hautkrebsscreening beim Arzt ergänzen bzw. unterstützen:

☐ Ja ☐ Nein ☐ Weiß nicht

Hautkrebs-Apps können das Hautkrebsscreening beim Arzt ersetzen:

☐ Ja ☐ Nein ☐ Weiß nicht

Glauben Sie, dass durch einen breiteren Einsatz von Hautkrebs-Apps das Auftreten von Hautkrebs reduziert werden kann?

☐ Ja ☐ Nein ☐ Weiß nicht

Glauben Sie, dass durch einen breiteren Einsatz von Hautkrebs-Apps Arztkosten eingespart werden können?

☐ Ja ☐ Nein ☐ Weiß nicht

Ich würde eher eine (Patienten-) Broschüre zum Thema Hautkrebs lesen statt eine App herunterzuladen.

☐ Ja ☐ Nein ☐ Weiß nicht

Ich würde eine Hautkrebs-App, die mir mein Arzt empfohlen hat, herunterladen.

☐ Ja ☐ Nein ☐ Weiß nicht

Haben Sie Bedenken bezüglich der unbefugten Weitergabe ihrer Informationen an Dritte bei der Benutzung einer Hautkrebs-App?

☐ Ja ☐ Nein ☐ Weiß nicht

Wünschen Sie mehr Aufklärung durch Ihren Hautarzt zu Hautkrebs-Apps?

☐ Ja ☐ Nein ☐ Weiß nicht

Angaben zu Ihrer Person:

Sind Sie männlich oder weiblich?

☐ Männlich ☐ Weiblich

Bitte geben Sie Ihr Alter an:

_________ Jahre

Was ist Ihr höchster erreichter Bildungsabschluss?

☐ Kein Schulabschluss
☐ Realschulabschluss
☐ (Fach-)Abitur
☐ Hauptschul-/Volksschulabschluss
☐ Sonderschulabschluss
☐ Hochschulabschluss

☐ Anderer Schulabschluss: ____________________

Sie sind nun am Ende des Fragebogens angelangt. Vielen Dank für Ihre Teilnahme.

Bitte werfen Sie den Fragebogen und Stift in Briefkasten vor der onkologischen Ambulanz ein!
